# Inhibition of Soluble Epoxide Hydrolase for Renal Health

**DOI:** 10.3389/fphar.2018.01551

**Published:** 2019-01-10

**Authors:** Jun-Yan Liu

**Affiliations:** ^1^Center for Nephrology and Metabolomics, Tongji University School of Medicine, Shanghai, China; ^2^Division of Nephrology, Shanghai Tenth Peoples Hospital, Tongji University School of Medicine, Shanghai, China

**Keywords:** chronic kidney disease, acute kidney disease, soluble epoxide hydrolase, epoxyeicosatrienoic acid, renal dysfunction

## Abstract

A soluble epoxide hydrolase (sEH) mediates the metabolism of epoxy fatty acids to form the corresponding vicinal diols, which are usually inactive or less active than the epoxide substrates. The sEH enzyme presents in many organs, including but not limited to the liver, heart, spleen, lung, and kidney. Here we summarized the changes in the expression and activity of sEH in multiple renal diseases, such as acute kidney injury (AKI), diabetic nephrology (DN), chronic kidney diseases (CKD), hypertension-mediated renal damage, and other renal dysfunctions. We also discussed the pharmacologic effects and the underlying mechanisms of sEH inhibition by using an inhibitor of sEH and/or the generic deletion of sEH on multiple renal diseases. We believe that sEH is a potential therapeutic target for renal dysfunction although the target disease needs further investigation.

## Introduction

**FIGURE 1:**
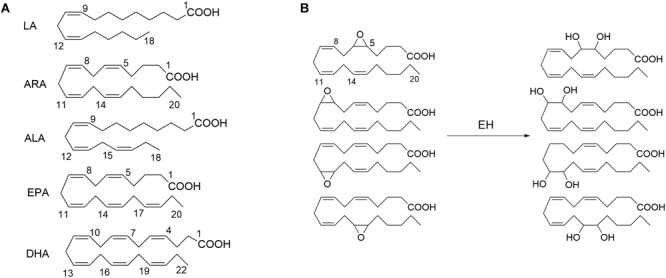
The exemplified polyunsaturated fatty acids **(A)** that are the common substrates for epoxide hydrolase (EH) and **(B)** four epoxide regioisomers of ARA that could be metabolized to form the respective diols in the presence of EH such as soluble epoxide hydrolase (sEH) and microsomal epoxide hydrolase (mEH). LA, linoleic acid [18:2 (n–6)]; ARA, arachidonic acid [20:4 (n–6)]; ALA, alpha-linolenic acid [18:3 (n–3)]; EPA, eicosapentaenoic acid [20:5 (n–3)]; and DHA, docosahexaenoic acid [22:6 (n–3)].

A soluble epoxide hydrolase (sEH), a member of epoxide hydrolases (EH) family, presents in almost all living organisms (Newman et al., 2005; Morisseau and Hammock, 2013). In humans, sEH is encoded by the gene *EPHX2* (Larsson et al., 1995; Sandberg and Meijer, 1996). The sEH has been well known to mediate the metabolism of epoxides to form the corresponding vicinal diols (Newman et al., 2005). The epoxids that could serve as the substrates for a sEH have been well documented previously, one member of which is epoxy fatty acids, such as the epoxy metabolites of PUFAs, including but not limited to linoleic acid [LA, 18:2 (n-6)], arachidonic acid [ARA, 20:4 (n-6)], alpha-linolenic acid [ALA,18:3 (n-3)], eicosapentaenoic acid [EPA, 20:5 (n-3)], and docosahexaenoic acid [DHA, 22:6 (n-3)] (Figure 1; Newman et al., 2005).

Many epoxy fatty acids are multifunctional mediators *in vivo* and *in vitro*. For example, EETs, the epoxide metabolites of ARA, are anti-inflammatory (Node et al., 1999), analgesic (Inceoglu et al., 2008), and EDHF (Campbell et al., 1996). However, the sEH-mediated diol metabolites of epoxide fatty acids are usually inactive or less active than their respective epoxide precursors (Newman et al., 2005; Morisseau and Hammock, 2013). The substrate selectivity for the sEH-mediated metabolism of epoxide fatty acids was reported previously (Zeldin et al., 1995; Deng et al., 2017). Although the active epoxy fatty acids could be degraded easily, the circulation and tissue levels of active epoxy fatty acids could be stabilized by both pharmacological interventions of an inhibitor of sEH and target gene disruption of *EPHX2*. Therefore, sEH inhibitors have been extensively reported to be anti-inflammatory, analgesic, anti-hypertensive, anti-fibrotic, cardioprotective and renoprotective, and other functions *in vivo* and *in vitro* (Imig and Hammock, 2009; Morisseau and Hammock, 2013; Sirish et al., 2013; Li et al., 2014; Fan et al., 2015; He et al., 2016).

**FIGURE 2:**
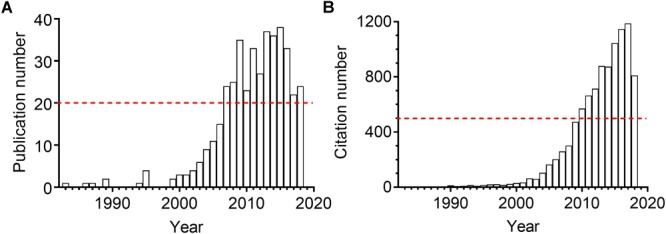
The annual publication numbers **(A)** and the resulting citation numbers **(B)** of the scientific paper regarding sEH and kidney. The results were generated by searching the Web of Science with the topic “sEH” combined the topic “kidney or renal” on October 1, 2018.

The sEH presents in almost all mammal organs, such as heart, liver, lung, spleen, intestine, stomach, brain, and urinary and developmental organs^1^. Kidneys, as the vital part of the urinary and excretory system, also express sEH. The expression and activity were reported upregulated in many kidney diseases for human and animals. Inhibition of sEH was therefore reported to be renoprotective in many renal diseases. As illustrated in Figure 2, the attention to the sEH and kidneys has been consistently rising in the last 10 years. Here we summarized the pathophysiological and pharmacological function of sEH in the onset, prevention, and treatment in multiple kidney-associated diseases. The underlying molecular mechanisms of sEH inhibition on renal diseases were also discussed in this review paper.

## The Presence and Localization of sEH in the Kidneys

The enzyme sEH was expressed in all the organs investigated including but not limited to liver, kidney, brain, stomach, intestines, pancreas, prostate, heart, lung, and skin^1^. The sEH has been reported to present in human and animal kidneys at both transcription and protein levels (Table 1). By using IHC staining, Enayetallah et al. (2004) reported the distribution and expression of sEH in an array of normal human tissues. They found that sEH was frequently expressed in the human kidneys (*n* = 15) with a high level in the renal proximal tubules but a low level in the renal distal tubules and meager presence in the glomeruli (Enayetallah et al., 2004). In a follow-up study, Enayetallah et al. (2006) found that the sEH is presented in the proximal and cytosolic compartments in hepatocytes and renal proximal tubules. Wang et al. (2013, 2018) also reported that sEH expressed in the renal tubules in the patients with IgAN and other glomerulonephritis. In contrast, Yu et al. (2004) reported the cellular localization of sEH in the human kidneys by examining the biopsies taken from the patients with multiple non-end-stage renal diseases (*n* = 8) and those without known renal disorders (*n* = 7). Yu et al. (2004) found that sEH was preferentially expressed in the renal vasculature, mostly in the smooth muscle layers of the arterial wall, while relatively low levels in the surrounding tubules. Also, Yu et al. (2004) reported that sEH expressed in the normal kidneys in a similar pattern to those in the diseased kidneys in the samples investigated. The inconsistent observations among these studies may be due to the different sampling locations of renal biopsies. In addition, the sEH was also reported to be present in the murine and rodent kidneys (Patel et al., 1986; Johansson et al., 1995). The presence of sEH in human and animal kidneys opens a possibility that sEH could be associated by multiple renal diseases.

**Table 1:** The location and co-location of sEH in kidneys from different species.

Localization/colocalization	Species	Detection methods	Reference
Proximal tubules	Human	Immunohistochemistry	Enayetallah et al., 2004
Peroxisomal and cytosolic compartments of renal proximal tubules	Human	Immunofluorescence	Enayetallah et al., 2006
Tubular epithelial cells	Human	Immunohistochemistry	Wang et al., 2018
Proximal tubular cells	Human	Immunohistochemistry	Wang et al., 2013
Vasculature	Human	Immunohistochemistry	Yu et al., 2004
Proximal tubular epithelial cells	Rat	Immunoblotting	Wang et al., 2013
Renal proximal tubular epithelial cells	Rat	Immunoblotting	Liang et al., 2015
Kidney	Rat	q-PCR, ELISA	
Kidney	Rat	q-PCR	Abramova et al., 2013
Kidney	Rat	q-PCR, immunoblotting	Chábová et al., 2018
Kidney	Rat	Immunoblotting	Cervenka et al., 2015
Kidney	Rat	cDNA microarray, immunoblotting	Seubert et al., 2005
Cortex	Rat	Immunoblotting	Imig et al., 2002; Walkowska et al., 2010
Microvessels and cortex	Rat	Immunoblotting	Zhao et al., 2004
Soluble fraction	Mice	Product diol	Patel et al., 1986
Kidney, podocytes	Mice	Immunoblotting, q-PCR, immunofluorescence	Bettaieb et al., 2017b,a
Kidney	Mice	Immunohistochemistry, immunoblotting	Chiang et al., 2015
Kidney	Mice	immunoblotting	Chen G. et al., 2012; Kim et al., 2014, 2015
Cortex	Mice	q-PCR, immunoblotting	Oguro et al., 2009; Jung et al., 2010; Luo et al., unpublished
Cortex	Mice	q-PCR	Roche et al., 2015

## Preclinical Studies of the Treatment of Renal Dysfunction by Regulation of sEH

Although there has been no drug clinically used as a sEH inhibitor yet, a large amount of pre-clinically experimental evidence supports that sEH may be a potential therapeutic target for several kidney-associated diseases, such as acute kidney injury (AKI), chronic kidney disease (CKD), diabetic nephrology (DN), and hypertension-associated kidney damage.

### Regulation of sEH for the Treatment of AKI

AKI is a common fatal disease in hospitals characterized by a sudden and sustained reduction in renal function with the phenotypes of an abrupt decrease in urine output and a dramatic increase in serum creatinine level. The mechanisms underlying the pathogenesis of AKI vary, including but not limited to ischemia/reperfusion, septic shock, toxicant exposure, and inflammation-caused decrease in kidney blood flow, resulting in the damage to renal tissues, and eventually renal dysfunction (Kusch et al., 2013; Persson, 2013; Glodowski and Wagener, 2015; Takaori and Yanagita, 2016), which involve the impairment in glomerulus and renal tubular epithelial cells (Kusch et al., 2013; Prieto-Garcia et al., 2016), and activation of NFκB and GSK-3β (Liu et al., 2012; Kusch et al., 2013; Deng et al., 2017).

**FIGURE 3:**
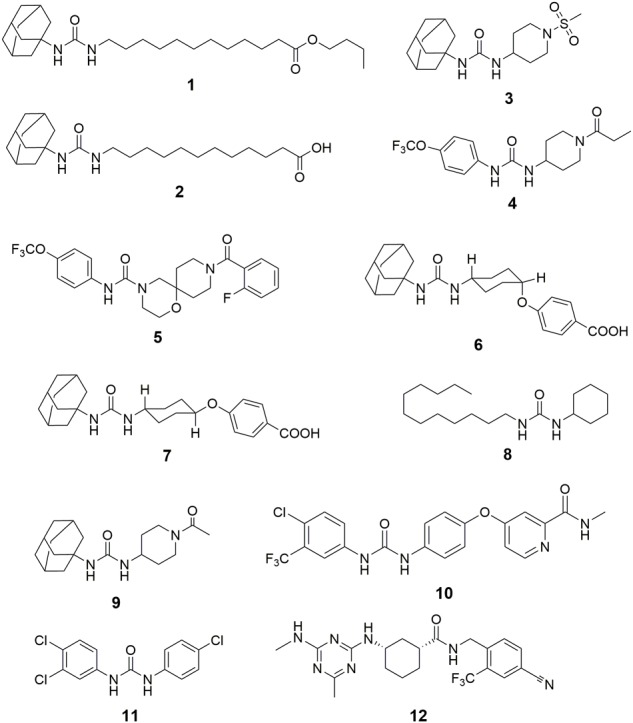
The chemical structures of the inhibitors of sEH summarized in this paper. 1, n-butyl ester of 12-(3-adamantan-1-yl-ureido)-dodecanoic acid (nbAUDA); 2, 12-(3-adamantan-1-yl-ureido)-dodecanoic acid (AUDA); 3, 1-adamantan-1-yl-3-(1-methylsulfonyl-piperidin-4-yl-urea) (AR9273); 4, 1-trifluoromethoxyphenyl-3-(1-propionylpiperidin-4-yl)urea (TPPU); 5, 9-(2-fluorobenzoyl)-N-[4-(trifluoromethoxy)phenyl]-1-oxa-4,9-diazaspiro[5.5]undecane-4-carboxamide; 6, *cis*-4-[4-(3-adamantan-1-yl-ureido)cyclohexyloxy]benzoic acid (*c*-AUCB); 7, *trans*-4-[4-(3-adamantan-1-yl-ureido)cyclohexyloxy]benzoic acid (*t*-AUCB); 8, 1-cyclohexyl-3-dodecylurea (CDU); 9, 1-(1-acetypiperidin-4-yl)-3-adamantanylurea (AR9281); 10, 4-[4-[[4-chloro-3-(trifluoromethyl)phenyl]carbamoylamino]phenoxy]-N-methyl-pyridine-2-carboxamide (Sorafenib); 11, 3-(4-Chlorophenyl)-1-(3,4-dichlorophenyl)urea (Triclocarban); 12, (1R,3S)-N-[4-cyano-2-(trifluoromethyl)benzyl]-3-[(4-methyl-6-(methylamino)-1,3,5-triazin-2-yl)amino] cyclohexane-1-carboxamide (GSK2256294).

Inhibition of sEH has been reported to attenuate the renal injury in multiple murine models of AKI. Although Hashimoto et al. (2015) reported that cisplatin administration decrease the activity and expression of sEH in the kidneys of male ddY mice, Parrish et al. (2009) found that subcutaneous (*s.c*.) injection of a sEH inhibitor n-butyl ester of 12-(3-adamantan-1-yl-ureido)-dodecanoic acid (nbAUDA, 1, Figure 3), a pro-drug of AUDA (Newman et al., 2005) significantly attenuated the renal injury in a C3H mice model of AKI caused by intraperitoneal (i.p.) injection of cisplatin, which was supported by the blood levels of urea nitrogen (BUN) and histological analysis of kidneys. Also, in a murine model of AKI caused by i.p. injection of cisplatin, Liu et al. reported that oral administration of another sEH inhibitor, 1-adamantan-1-yl-3-(1-methylsulfonyl-piperidin-4-yl-urea) (AR9273, 3) that is structurally different from AUDA, markedly attenuated renal injury, which was supported by the serum levels of urea nitrogen and creatinine, and histological evidence of renal tubular damage and neutrophil infiltration (Liu et al., 2012, 2013). Also, the renoprotective effects of AR9273 were consistent with those in the sEH knockout mice (Liu et al., 2012). It should be noted that the sEH inhibitors were administered 1 day before cisplatin treatment in both studies. Therefore, the renoprotective effects of sEH inhibitors are prophylactic rather than therapeutic effects.

In a murine model of AKI caused by ischemia/reperfusion, Deng et al. (2017) reported that administration of a sEH inhibitor, TPPU, 4 attenuated renal injury, which was supported by plasma creatinine, survival time, and histological analysis. In contrast, Zhu et al. (2016) also reported that sEH knockout exacerbated renal injury caused by ischemia/reperfusion in a murine model. Interestingly, Zhu et al. (2016) found that the renal level of 20-HETE and one of its synthetic enzymes, Cyp4a12a, were increased significantly in sEH knockout mice when compared with those of wildtype mice. 20-HETE is a potent nephrotoxic compound, which may abate the renoprotective effects of increased EETs caused by sEH deficiency.

In a murine model of AKI caused by lipopolysaccharide (LPS), Bettaieb et al. (2017a) reported that podocyte-specific sEH deficiency ameliorated the LPS-caused mice renal injury, which was supported by the favorable changes in proteinuria, BUN, and renal mRNA levels and serum levels of inflammatory cytokines. In addition, the *in vivo* renal protective effect of podocyte-specific sEH knockout was further supported by the *in vitro* data from the treatment of a sEH inhibitor TPPU to the E11 murine podocytes (Bettaieb et al., 2017a).

### Inhibition of sEH for the Treatment of CKD

CKD is one of the top public health problems and leading diseases for all-cause mortality globally (Xie et al., 2018). The putative mechanisms regarding the pathogenesis and progression of CKD have been well documented previously (Fogo, 2007; Malyszko, 2010; Eddy et al., 2012; Yang et al., 2017; Cheung et al., 2018; Mihai et al., 2018; Moradi and Vaziri, 2018; Rossi et al., 2018). Briefly, both generic factor-caused genital abnormality of renal development, and other factors (e.g., inflammation, hypertension, diabetes, dyslipidemia, disorder of cytokines and growth factors, proteinuria, podocyte loss, etc.) can cause irreversible scarring in kidney, resulting in progressive renal fibrosis, and eventually end-stage renal disease, which needs renal replacement therapy, such as hemodialysis and renal transplantation. The progression of CKD has been reported to be associated with the damage to renal glomerulus and renal epithelial cells, accelerating the cell migration and epithelial-mesenchymal transition, and repressing the proliferation of podocytes, which may be mediated by angiotensin-II, PPAR-γ, AMPK, CYPs, and sEH (Fogo, 2007; Malyszko, 2010; Decleves et al., 2011; Kim et al., 2014; Fan et al., 2015).

Inhibition of sEH by both pharmacological interventions with a sEH inhibitor and target gene disruption of sEH has been reported to attenuate the renal injury in several murine and rodent models. Kato et al. (2014) reported that a potent sEH inhibitor, 9-(2-fluorobenzoyl)-N-[4-(trifluoromethoxy)phenyl]-1-oxa-4,9-diazaspiro[5.5]undecane-4-carboxamide (Node et al., 1999), amelio-rated renal injury in a rat model of anti-glomerular basement membrane glomerulonephritis evidenced by time-dependently reducing the rat serum creatinine. Liang et al. (2015) reported that the treatment of AUDA abated the proteinuria-induced epithelial-mesenchymal transition *in vivo* and *in vitro*.

By using a rodent model of CKD caused by 5/6 nephrectomy (5/6 NX) in Ren-2 transgenic rats, Kujal et al. (2014) reported that the treatment of a sEH inhibitor, *cis*-4-[4-(3-adamantan-1-yl-ureido)cyclohexyloxy]benzoic acid (*c*-AUCB, 6) attenuated the renal and cardiac injuries of the diseased rats by modification of the survival rate, blood pressure, cardiac hypertrophy, proteinuria, degree of glomerular and tubulointerstitial injury, and glomerular volume toward the normative status. In addition, Chábová et al. (2018) reported that co-administration of *c*-AUCB with a standard renin-angiotensin system (RAS) blockade resulted in additional therapeutic effects in the improvement of rat survival rate, reduce in albuminuria, glomerular and tubulointerstitial injury when compared with the standard RAS blockade alone.

In a murine model of obstructive nephropathy caused by UUO surgery, Kim et al. (2014) reported that the deficiency of sEH abolished the renal interstitial fibrosis and inflammation. Consistently, Chiang et al. (2015) found that renal expression of sEH protein was increased in the UUO-treated mice when compared with the sham mice. In addition, the target gene disruption of sEH abated the UUO-caused renal injury, such as hydronephrosis, renal tubular injury, inflammation, and fibrosis. Also, oral administration of a sEH inhibitor, *trans*-4-[4-(3-adamantan-1-yl-ureido)cyclohexyloxy]benzoic acid (*t* -AUCB, 7) resulted in the similar fibro-protective and anti-inflammatory effects to sEH gene knockout (Kim et al., 2014, 2015). Interestingly, Yang et al. (2017) reported that treatment of AUDA resulted in similar results to *t*-AUCB in a UUO-induced mice model of renal fibrosis.

In addition, Huang et al. (2007) reported that inhibition of sEH by administration of a sEH inhibitor AUDA attenuated the renal injury by regulating the mean arterial pressure, renal vascular resistance, and glomerular filtration rate, and renal blood flow for the obese rats toward normative status. By using a target metabolomic analysis, Luo et al. (unpublished) found that renal sEH was upregulated at both transcription and protein levels time-dependently upon the challenge of high-fat diet (HFD) feeding, which was associated with the progression of renal injury. In addition, inhibition of sEH by TPPU attenuated the HFD-induced renal injury by, at least in part, activation of the Ampk-mediated macro-autophagy and Pax2-mediated chaperone-mediated autophagy.

It is worth noting that not all the study showed the favorite effect of sEH inhibition on kidney diseases. Jung et al. reported in a mice model of chronic renal failure caused by 5/6-nephrectomy that c-TUCB (Inceoglu et al., 2008) failed in lowering blood pressure and even aggravated albuminuria when compared with the placebo controls (El-Sherbeni et al., 2013). The authors thought that this unfavorite effect may be due to the shifts of arachidonic metabolism into lipoxygenase pathway by sEH inhibition.

### Inhibition of sEH for the Treatment of Diabetic Nephrology

We separated DN from CKD in the “Inhibition of sEH for the treatment of CKD” since DN is a leading cause of CKD and end-stage renal disease (Lin and Sheu, 2014). DN causes primary renal damage to its microvascular system including glomerular capillaries, influent and affluent arteries at the beginning, and most leading to end-stage renal disease over time (Remuzzi et al., 2006; Afsar and Elsurer, 2017), which may involve the activation of some transcription factors (e.g., activator protein 1, cAMP-response element-binding protein, nuclear factor of activated T cells, NF-κB, stimulating protein 1, and upstream stimulatory factor 1) and may be regulated by some signal pathways such as mTOR, AMPK, GSK-3β, and Deptor 2 (Mariappan, 2012).

Chen G. et al. (2012) reported that sEH deficiency maintained the renal function in a murine model of STZ-induced DN. Compared with the wildtype diabetic mice, sEH-deficient mice resulted in significantly decreased levels of plasma Hb A_1c_ and creatinine, BUN and urinary microalbumin excretion. Bettaieb et al. (2017b) reported that renal sEH protein was upregulated in the mice under HFD and STZ-induced hyperglycemia. In addition, podocyte-specific sEH deficiency preserved renal function *in vivo* and *in vitro* via modulation of the renal endoplasmic reticulum (ER) stress, inflammation, fibrosis, and autophagy toward normative conditions, which was further supported by the *in vitro* data from the pharmacological intervention of sEH with the sEH inhibitor TPPU (Bettaieb et al., 2017b). Katary et al. (2017) reported that in a rodent model of STZ-induced DN, the treatment with *t*-AUCB attenuated the renal injury by reducing glomerular albumin permeability, albumin, and nephrin excretion levels and restoring the decrease in glomerular α3 integrin and nephrin expression in diabetic rats. However, this beneficial effect of sEH inhibition was unable to be enhanced by co-administration with meloxicam, a cyclooxygenase inhibitor (Katary et al., 2017).

### Inhibition of sEH for the Attenuation of Hypertension-Associated Kidney Disorders

Hypertension is the second leading cause of end-stage renal disease after diabetes in the United States (U.S. Renal Data System, 2009). Although the susceptibility to hypertension-associated renal injury differs significantly in various populations, a consensus is that hypertension causes damage to glomerular arteries and capillary, and endothelial cells, leading to the injuries to glomerular filtration barrier and podocytes, eventually renal dysfunction, which may be manipulated by renin-angiotensin–aldosterone system, reactive oxidative species, endothelial dysfunction, and genetic and epigenetic factors (Mennuni et al., 2014).

Hypertension is a risk factor for the development of renal dysfunction. The renal sEH was upregulated in hypertensive status. By using a gene microarray analysis, Seubert et al. (2005) reported that sEH was significantly upregulated in the kidneys of spontaneously hypertensive rats (SHR) when compared with the non-hypertensive Wistar-Kyoto (WKY) rats. Also, Abramova et al. (2013, 2017) found that the renal mRNA level and protein concentration of sEH were increased at in the inherited stress-induced arterial hypertension (ISIAH) rats when compared with the normative Wistar Albino Glaxo (WAG) rats. Tain et al. (2015) reported that renal sEH was upregulated in dexamethasone- and a high fructose-induced rodent model of programmed hypertension. These data suggest the possibility that inhibition of sEH could protect the kidney from injury associated with hypertension.

Zhao et al. (2004) reported that chronic administration of a sEH inhibitor 1-cyclohexyl-3-dodecyl urea (CDU, 8) significantly attenuated renal injury in an angiotensin-induced rodent model of hypertension, which was supported by the observation that CDU treatment maintained the renal vasculatures and glomerulus toward normative status. Imig et al. (2005) reported that administration of AUDA significantly lowered blood pressure and protected renal damage by decreasing the urinary microalbumin excretion in the rodent models of hypertension induced by both normal-salt angiotensin and high-salt angiotensin. Imig et al. also reported that another sEH inhibitor AR9281 attenuated glomeruli injury and reduced renal inflammation in the angiotensin-induced hypertensive rats (Imig et al., 2009). Olearczyk et al. (2009) reported that treatment with AUDA protected kidneys from glomerular and tubular damage in spontaneously diabetic Goto-Kakizaki rats induced by angiotensin II with high salt diet. Sporkova et al. (2011) reported that treatment of *c*-AUCB improved renal function by maintaining the renal blood flow toward normative status while exhibiting an anti-hypertensive effect in the 2-kidney 1 clip hypertensive rats. Imig et al. (2012) reported that *t*-AUCB treatment reversed the increase in urinary levels of albumin and kidney injury marker-1 (KIM-1) in the spontaneously hypertensive obese rats. In addition, co-administration of *t*-AUCB with rosiglitazone, an agonist of PPARγ, resulted in additive reno-protection (Imig et al., 2012). Cervenka et al. (2015) reported that in an aorto-caval fistula-induced Ren-2 transgenic hypertensive rats model of congestive heart failure (CHF), *c*-AUCB treatment improved rats survival rate and increased renal blood flow, glomerular filtration rate and fractional sodium excretion.

## Association of sEH Polymorphisms With Kidney-Associated Diseases

Several sEH polymorphisms, such as Lys55Arg, rs41507953 (K55R), rs751141 (R287Q), and rs1042032, were reported to correlate with several renal-associated diseases. Based on current data, lower sEH activity is associated with the improved renal function. Shuey et al. (2017) reported that sEH Lys55Arg polymorphism was associated with an increased incidence of AKI following cardiac surgery in patients without preexisting CKD. Also, the sEH activity that was characterized by the ratio of DHOMEs to EpOMEs was increased in sEH 55Arg variant carriers when compared with the investigated wildtype carriers.

Lee et al. (2011) investigated the association of three single nucleotide polymorphisms [SNPs, rs41507953 (K55R), rs751141 (R287Q), and rs1042032] of sEH with IgAN progression in a retrospective cohort including 401 IgAN patients and 402 normal healthy controls. They reported that the patients carrying the variant allele (A) of rs751141 (R287Q) were associated with a better kidney survival (*P* < 0.001) and a lower sEH activity (*P* < 0.05) than those with the wildtype allele (G) (Lee et al., 2011). Also, Wang et al. (2013, 2018) reported that the level of sEH expression correlated with proteinuria and infiltration of macrophages positively in the IgAN patients and other glomerulonephritis.

Dreisbach et al. (2005) conducted a case-control study to investigate the possible correlation of multiple SNPs in sEH (sEH-Q and sEH-RR) and CYP 2C8, 2C9, and 2J2 with an increased risk of hypertension in African American individuals. They found that these SNPs are not associated with the increased risk of hypertension in the African American population (Dreisbach et al., 2005). Ma et al. (2018) reported that the A allele of an exonic polymorphism in sEH rs751141 correlated with the incidence of DN in the Chinese T2D population negatively, which could be modulated by homocysteine level status.

Gervasini et al. (2015) reported that the renal transplant recipients who carried the rs1042032GG genotype in sEH were associated with the significantly lower estimated glomerular filtration rate and higher serum creatinine levels on year after grafting compared with those with wildtype A-allele. This was consistent with the study by Lee et al. (2008), in which the presence of the rs1042032 AA genotype in sEH was reported to correlate with a protective role for allograft function for the kidney transplant recipients.

## Molecular Mechanisms Underlying the Regulation of sEH for Reno-Protection

The sEH has been reported to be upregulated by both endogenous and exogenous factors at both mRNA and protein levels. Wang et al. (2013, 2018) reported that the expression of sEH was increased in renal tissues in the patients with glomerulonephritis when compared with the control renal tissues by IHC analysis. Both mRNA and protein levels of sEH were found higher in the kidneys of SHR than those of the control WKY rats (Yu et al., 2000; Koeners et al., 2011). The sEH was also upregulated in the renal cortex by angiotensin-II, two-kidney-one-clip, and a HFD (Imig et al., 2002; Zhao et al., 2004; Kopkan et al., 2012; Luo et al., unpublished).

An sEH can be down-regulated by generic target gene disruption of *Ephx2* and chemical knockdown by various sEH inhibitors. In most cases, sEH is believed to be inhibited at protein level primarily due to the formation of the hydrogen bonds between the inhibitors and the active sites Tyr461 and Tyr385 in both human and mammal sEH enzymes (Argiriadi et al., 1999, 2000; Gomez et al., 2004). Recently, Luo et al. (unpublished) found that an sEH inhibitor TPPU could inhibit murine sEH transcriptionally.

**FIGURE 4:**
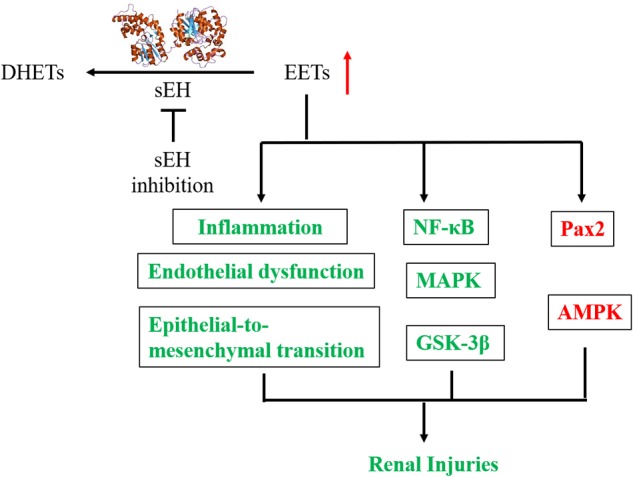
A putative mechanic summary of reno-protection of sEH inhibition. The items highlighted with red mean increased while those with green mean decreased caused by sEH inhibition with a chemical inhibitor or generic disruption.

The mechanisms underlying the reno-protection of sEH inhibition are summarized in Figure 4. The renoprotective effects of sEH inhibition are mostly believed to be ascribed to the increased renal levels of EETs resulted from pharmacological interventions with a sEH inhibitor or genetic disruption of sEH based on two main facts. The one is that the renal and/or circulation levels EETs or the intrarenal ratio of EETs to DHETs were hypothesized and observed to be increased following sEH inhibition (Zhao et al., 2004; Huang et al., 2007; Kim et al., 2014, 2015; Kujal et al., 2014; Deng et al., 2017; Luo et al., unpublished). The other is that EETs have been investigated to be the active mediators, which could result in the protection of kidneys from multiple injuries. In addition to inhibition of sEH, the metabolic pathway of EETs, upregulation of CYP2C and/or 2J, the biosynthetic pathways of EETs, is an alternative approach to stabilize the EETs level *in vivo.* Upregulation of Cyp2J was previously reported to protect the kidneys function in the 5/6-nephrectomized rat (Zhao et al., 2012), which supports that the reno-protection of EETs.

The biochemical functions of EETs have been well documented in several review papers (Spector et al., 2004; Spector and Norris, 2007; Wang and DuBois, 2012; Imig, 2015). EETs were reported to be anti-inflammatory via modulation of the NFκB/IκBK signaling pathway (Node et al., 1999). Reno-protective effects of sEH inhibition, such as anti-inflammatory and fibro-protective, were reported to be involved in the modulation of NFκB signaling pathway and cytokine orchestra toward inflammation resolution in several animal studies (Liu et al., 2012; Kim et al., 2014, 2015; Bettaieb et al., 2017a). Also, EETs are potent EDHF (Campbell et al., 1996; Archer et al., 2003). Inhibition of sEH was reported to protect the kidney from the multiple injuries associated with the improvement of the renal microvessel dilation and renal blood flowing (Sporkova et al., 2011; Cervenka et al., 2015).

Epoxyeicosatrienoic acids have been reported to induce cell growth and inhibit the cell apoptosis (Dhanasekaran et al., 2006; Nieves and Moreno, 2007). EETs were found to protect pig kidney proximal tubule LLC-PK1 cells from cisplatin-induced p38 MAPK activation, and Bax mitochondrial trafficking (Liu et al., 2013), which was further supported by that inhibition of sEH by AR9273 treatment and sEH-deficiency significantly attenuated cisplatin-induced murine renal injury via inhibition of the p38 MAPK phosphorylation (Liu et al., 2013). The renal podocyte-specific sEH deficiency ameliorated murine kidney injury associated with decreased LPS-induced NF-κB and MAPK activation and attenuated endoplasmic reticulum stress (Bettaieb et al., 2017a). EETs were also reported to inhibit the apoptosis of renal tubular epithelial cells via, at least in part, the modulation of the phosphorylation of GSK-3β, which was also supported by the *in vivo* data from TPPU treatment in a murine model of AKI (Deng et al., 2017). Also, AUDA treatment was reported to attenuate renal injury by modulation of the PI_3_K-Akt-GSK-3β signaling pathway (Liang et al., 2015). Also, sEH gene inhibition and exogenous EETs significantly protected HK-2 cells from TNF-induced apoptosis *in vitro* associated with activation of the PI3K-Akt-NOS3 and AMPK signaling cascades (Chen G. et al., 2012).

Recently, Luo et al. (unpublished) found that EETs protected the murine renal mesangial cells and tubular epithelial cells from palmitic acid-induced injury by activation of Ampk and Pax2, which was supported by the *in vivo* data from the TPPU treatment to the mice feeding on a HFD.

In addition to EETs, other epoxy fatty acids were also reported to contribute to the renoprotective role of sEH inhibition. 19(20)-epoxydocosapentaenoic acid, the principal epoxy metabolite of DHA that could also be stabilized by sEH inhibition, was found to mitigate the renal fibrosis in a mouse model of experimental UUO-induced renal fibrosis by decreasing renal epithelial-to-mesenchymal transition (Sharma et al., 2016).

Besides sEH, microsomal EH (mEH), encoded by *EPHX1*, has the similar function to sEH in mediating the hydrolysis of epoxy fatty acids (Newman et al., 2005; Rawal et al., 2009). However, mEH was found to modified slightly while sEH was up-regulated significantly in renal microvessels by the treatment of angiotensin-II infusion (Zhao et al., 2004). Recently, mEH was found to be decreased significantly while sEH altered non-significantly at mRNA level in the blood cells of the uremic patients when compared with healthy controls (Hu et al., 2018). The pathophysiological and pharmacological role of mEH in kidney diseases and other disorders needs further investigation.

## Clinical Studies of sEH Inhibitors

As far as we know, there is no clinical drug used as a sEH inhibitor. However, some sEH chemical inhibitors failed in, or are under, or will be tested in clinical trials. In addition, some clinically used drugs or human used agents were found to be the potent sEH inhibitors although they have their specific mode of action other than sEH inhibition. For example, sorafenib (Imig and Hammock, 2009) is a clinical drug for the treatment of advanced renal cell carcinoma, advanced hepatocellular carcinoma, and radioactive iodine resistant advanced thyroid carcinoma as an inhibitor of multi-kinase, such as VEGFR, PDGFR, and Raf family kinases (Ng and Chen, 2006; Wilhelm et al., 2008; Smalley et al., 2009; Iyer et al., 2010). However, since sorafenib has the *N,N*′-disubstituted substructure that is similar to many potent sEH inhibitors (Figure 3), it was found to be a potent sEH inhibitor (Liu et al., 2009). Also, triclocarban (He et al., 2016) has been widely used as an anti-microbial agent in various personal care products (Brausch and Rand, 2011; Bu et al., 2013), is also a potent inhibitor of sEH (Liu et al., 2011).

In addition, two sEH inhibitors, AR9281 and GSK2256294, finished the phase I clinical trials. AR9281 showed well-tolerated at a single oral dose (10–1000 mg) and multiple doses (100–400 mg every 8 h) during the test period (1 week) and dose-dependent blood drug concentration and activity to inhibit the blood sEH with 90% or greater inhibition. This study suggested a twice-daily or thrice-daily dosing regimen for the patients (Chen D. et al., 2012). GSK2256294 was also well-tolerated at the tested doses. Plasma drug concentration increased at a dose-related manner with a half-life of 25–43 h. The activity of GSK2256294 to inhibit sEH was dose-dependent, from an average of 41.9% (2 mg) to 99.8% (20 mg) (Lazaar et al., 2016).

Wang et al. (2018) reported that renal sEH expression was increased in patients with IgA nephrology. Before that, Wang et al. (2013) reported that renal sEH expression was upregulated in the patients with glomerulonephritis including IgA nephrology. These human data suggest that sEH may be a therapeutic target for some renal diseases like IgA nephrology. However, Hu et al. (2018) reported that the mEH (EPHX1) was downregulated while sEH was changed slightly in the whole blood cells of the uremic patients when compared with healthy controls. Therefore, further studies are needed to optimize a target renal disease for the follow-up clinical study of a sEH inhibitor.

## Conclusion

In summary, sEH may be a promising therapeutic target for the prevention and treatment of renal disorders. Also, various small molecular sEH inhibitors were synthesized in several laboratories with favorable pharmacokinetics and pharmacodynamics in multiple animals and humans. To move sEH inhibitors toward clinical use, the next step should focus more on the functional investigation of sEH in the pathology and pathophysiology of the renal dysfunction by using human samples to target a renal disease for systemic evaluation of the pharmacological and toxicological effects of sEH inhibitors.

## Author Contributions

J-YL designed and wrote the paper.

## Conflict of Interest Statement

The author declares that the research was conducted in the absence of any commercial or financial relationships that could be construed as a potential conflict of interest.

## Footnotes

1 https://www.proteinatlas.org

## Abbreviations

AKI: acute kidney injury
ALA: alpha-linolenic acid
AR9273: 1-adamantan-1-yl-3-(1-methylsulfonyl-piperidin-4-yl-urea)
ARA: arachidonic acid
AUDA: 12-(3-adamantan-1-yl-ureido)-dodecanoic acid
BUN: blood urea nitrogen
*c*-AUCB: *cis*-4-[4-(3-adamantan-1-yl-ureido)cyclohexyloxy]benzoic acid
CDU: 1-cyclohexyl-3-dodecyl urea
CKD: chronic kidney disease
CYP: cytochrome P450
DHA: docosahexaenoic acid
DHOME: dihydroxyoctadecanoic acid
DN: diabetic nephrology
EDHF: endothelium-derived hyperpolarizing factor
EET: epoxyeicosatrienoic acid
EH: soluble epoxide hydrolase
EPA: eicosapentaenoic acid
EpOME: epoxyoctadecanoic acid
GBM: glomerular basement membrane
20-HETE: 20-hydroxyeicosatetraenoic acid
IgAN: IgA nephropathy
IHC: immunohistochemical or immunohistochemistry
LA: linoleic acid
nbAUDA: n-butyl ester of 12-(3-adamantan-1-yl-ureido)-dodecanoic acid
PUFA: polyunsaturated fatty acid
SNP: single nucleotide polymorphism
STZ: streptozotocin
*t*-AUCB: *trans*-AUCB
TPPU: 1-trifluoromethoxyphenyl-3-(1-propionylpiperidin-4-yl)urea
UUO: unilateral ureteral obstruction
